# Analysis of the Genomics and Mouse Virulence of an Emergent Clone of Streptococcus dysgalactiae Subspecies *equisimilis*

**DOI:** 10.1128/spectrum.04550-22

**Published:** 2023-03-27

**Authors:** Stephen B. Beres, Randall J. Olsen, S. Wesley Long, Jesus M. Eraso, Sarrah Boukthir, Ahmad Faili, Samer Kayal, James M. Musser

**Affiliations:** a Laboratory of Molecular and Translational Human Infectious Disease Research, Center for Infectious Diseases, Department of Pathology and Genomic Medicine, Houston Methodist Research Institute and Houston Methodist Hospital, Houston, Texas, USA; b Department of Pathology and Laboratory Medicine, Weill Cornell Medical College, New York, New York, USA; c Department of Microbiology and Immunology, Weill Cornell Medical College, New York, New York, USA; d CHU de Rennes, Service de Bacteriologie-Hygiène Hospitalière, Rennes, France; e INSERM, CIC 1414, Rennes, France; f Université Rennes 1, Faculté de Médecine, Rennes, France; g Université Rennes 1, Faculté de Pharmacie, Rennes, France; h Chemistry, Oncogenesis, Stress, and Signaling, INSERM 1242, Rennes, France; Michigan State University

**Keywords:** *Streptococcus dysgalactiae*, genomics, pathogenesis, emerging clone

## Abstract

Streptococcus dysgalactiae subsp. *equisimilis* is a bacterial pathogen that is increasingly recognized as a cause of severe human infections. Much less is known about the genomics and infection pathogenesis of S. dysgalactiae subsp. *equisimilis* strains compared to the closely related bacterium Streptococcus pyogenes. To address these knowledge deficits, we sequenced to closure the genomes of seven S. dysgalactiae subsp. *equisimilis* human isolates, including six that were *emm* type *stG62647*. Recently, for unknown reasons, strains of this *emm* type have emerged and caused an increasing number of severe human infections in several countries. The genomes of these seven strains vary between 2.15 and 2.21 Mbp. The core chromosomes of these six S. dysgalactiae subsp. *equisimilis stG62647* strains are closely related, differing on average by only 495 single-nucleotide polymorphisms, consistent with a recent descent from a common progenitor. The largest source of genetic diversity among these seven isolates is differences in putative mobile genetic elements, both chromosomal and extrachromosomal. Consistent with the epidemiological observations of increased frequency and severity of infections, both *stG62647* strains studied were significantly more virulent than a strain of *emm* type *stC74a* in a mouse model of necrotizing myositis, as assessed by bacterial CFU burden, lesion size, and survival curves. Taken together, our genomic and pathogenesis data show the strains of *emm* type *stG62647* we studied are closely genetically related and have enhanced virulence in a mouse model of severe invasive disease. Our findings underscore the need for expanded study of the genomics and molecular pathogenesis of S. dysgalactiae subsp. *equisimilis* strains causing human infections.

**IMPORTANCE** Our studies addressed a critical knowledge gap in understanding the genomics and virulence of the bacterial pathogen Streptococcus dysgalactiae subsp. *equisimilis*. S. dysgalactiae subsp. *equisimilis* strains are responsible for a recent increase in severe human infections in some countries. We determined that certain S. dysgalactiae subsp. *equisimilis* strains are genetically descended from a common ancestor and that these strains can cause severe infections in a mouse model of necrotizing myositis. Our findings highlight the need for expanded studies on the genomics and pathogenic mechanisms of this understudied subspecies of the Streptococcus family.

## INTRODUCTION

Streptococcus dysgalactiae subsp. *equisimilis* is a bacterial pathogen that causes diverse infections in humans; in recent years, it has been reported as the cause of increasing numbers of severe invasive infections (1–15). At the species level, S. dysgalactiae subsp. *equisimilis* is most closely related to Streptococcus pyogenes, a common cause of human infections, such as pharyngitis, skin and soft tissue infections, bacteremia, and severe invasive infections including necrotizing fasciitis, -myositis, and -pneumonia. Although generally perceived as less virulent, S. dysgalactiae subsp. *equisimilis* causes a spectrum of noninvasive and invasive human infections similar to that of S. pyogenes. S. dysgalactiae subsp. *equisimilis* is an increasingly recognized cause of serious invasive infections, and some strains have been documented to be resistant to beta-lactam antibiotics (16), a concerning development that may lead to substantial treatment difficulties and public health problems.

Compared to S. pyogenes, relatively few studies of the genomics and molecular pathogenic mechanisms of S. dysgalactiae subsp. *equisimilis* have been conducted, and thus much remains to be learned. For example, although there are 41,249 publicly available whole-genome sequencing run files for S. pyogenes, for S. dysgalactiae subsp. *equisimilis* there are only 648 (NCBI Sequence Read Archive as of 8 September 2022). Similarly, there are 2,223 publicly available S. pyogenes whole-genome assemblies with 257 closed, whereas for S. dysgalactiae subsp. *equisimilis* there are only 67 with 21 closed (NCBI Microbial Genome Database as of 8 September 2022). On average, the genome sequence of S. pyogenes strains is approximately 1.8 Mbp. In contrast, the genomes of S. dysgalactiae subsp. *equisimilis* strains are larger, averaging approximately 2.1 Mbp (8, 9, 17–24).

Similar to the relative lack of information about S. dysgalactiae subsp. *equisimilis* genomics, very little is known about the molecular basis of S. dysgalactiae subsp. *equisimilis* pathogenesis. S. dysgalactiae subsp. *equisimilis* strains share extensive gene content with S. pyogenes (25), but very few S. dysgalactiae subsp. *equisimilis* genes have been shown unambiguously to encode virulence factors experimentally proven to participate in pathogenesis based on analysis of isogenic mutant strains (19, 26–31). Also compared to S. pyogenes, only very limited pathogenesis work has been conducted with animal infection models, including necrotizing soft tissue infections (19, 27, 28, 30–32). Thus, in the aggregate, an understanding of S. dysgalactiae subsp. *equisimilis* pathogenic capacity is severely hampered by basic biology knowledge deficits, including population genomic diversity, gene expression, regulatory networks, virulence factors, and molecular interactions with the host.

The work presented here had two goals. First, we sought to investigate the genomics of *emm* type *stG62647* S. dysgalactiae subsp. *equisimilis* strains cultured from patients with infections in Brittany, France. In recent years, strains of this *emm* type have been an increasingly important cause of severe infections in several geographic areas (7, 9, 11, 13, 33–36). The *emm* type *stG62647* strains have been reported to cause an increased number of osteoarticular infections in humans compared to other S. dysgalactiae subsp. *equisimilis emm* types, although the cause is unknown (33, 37). Second, we sought to evaluate the relative virulence of the emergent *stG62647* lineage by comparing two clonally related *stG62647* strains with a genetically divergent strain of *emm* type *stC74a* using an established mouse model of necrotizing myositis (38–42). Our results provided important new information about the genetic relatedness and enhanced virulence of this emergent clone of S. dysgalactiae subsp. *equisimilis* and delineated a framework for expanded genomics and molecular pathogenesis analyses of understudied S. dysgalactiae subsp. *equisimilis* strains.

## RESULTS AND DISCUSSION

### Overview of genomes.

To establish a genetic foundation for investigating the molecular pathogenic capacity of S. dysgalactiae subsp. *equisimilis* strains to cause disease, the genomes of seven human infection isolates collected in French Brittany between 2010 and 2018 were determined by a combination of Illumina paired-end short-read and Oxford Nanopore long-read sequencing. The seven isolates included six group C strains of *emm* type *stG62647* (MGCS35823, MGCS35922, MGCS35957, MGCS36044, MGCS36083, and MGCS36089), which were selected because strains with these characteristics have recently emerged in multiple countries as a cause of increased numbers of severe infections (9, 36, 43). One group G *stC74a* strain (MGGS36055) was selected as a comparator, because strains with these characteristics have been reported to be an abundant cause of human infections in multiple epidemiological studies (7, 11, 15, 34, 44–46). The depth of short-read sequencing coverage averaged 587-fold (range, 204- to 1,539-fold) and long-read coverage averaged 94-fold (range, 21- to 239-fold), for a combined average of 681-fold (range, 337- to 1,560-fold) (see Table S1 in the supplemental material). The assembled and closed genomes had an average size of 2,196,360 bp (range, 2,151,694 to 2,233,554) with an average 2,130 protein-encoding genes (range, 2,075 to 2,209) and an average G+C content of 39.4% (range, 39.3% to 39.6%) (Table 1 and Table S2). Although some closed S. dysgalactiae subsp. *equisimilis* genomes differed in their overall architecture due to large regions of sequence inversion (for example, NCTC7136 versus ATCC 12394), these seven genomes were all colinear and had the same chromosomal architecture as reference strain ATCC 12394. These genome size and composition characteristics are consistent with those of the 21 publicly available closed S. dysgalactiae subsp. *equisimilis* genomes (Table S3). Consistent with the close species genetic relatedness, 1,280 of the 2,075 (61.7%) coding DNA sequences (CDSs) for the genome of strain MGCS36044 had a reciprocal Blastp best hit with a CDS of S. pyogenes *emm1* strain MGAS2221 genome (GenBank accession number GCA_012572265.1).

**TABLE 1:** Strains and closed genome characteristics

Strain	Yr	Invasive	Group antigen	MLST	*emm* type	Size (bp)	CDSs
MGCS35823	2015	Pos	C	20	*stG62647*	2,189,610	2,140
MGCS35922^*a*^	2016	Neg	C	20	*stG62647*	2,223,912	2,145
MGCS35957	2017	Neg	C	20	*stG62647*	2,233,554	2,209
MGCS36044	2018	Pos	C	20	*stG62647*	2,153,748	2,075
MGCS36083	2018	Pos	C	20	*stG62647*	2,212,841	2,140
MGCS36089	2018	Pos	C	20	*stG62647*	2,151,694	2,077
MGGS36055	2018	Pos	G	17	*stC74a*	2,204,518	2,126

a Strain MGCS35922 had additional gene content of 47,051 bp that assembled extrachromosomally.

### Regions of difference and putative mobile genetic elements.

**FIG 1:**
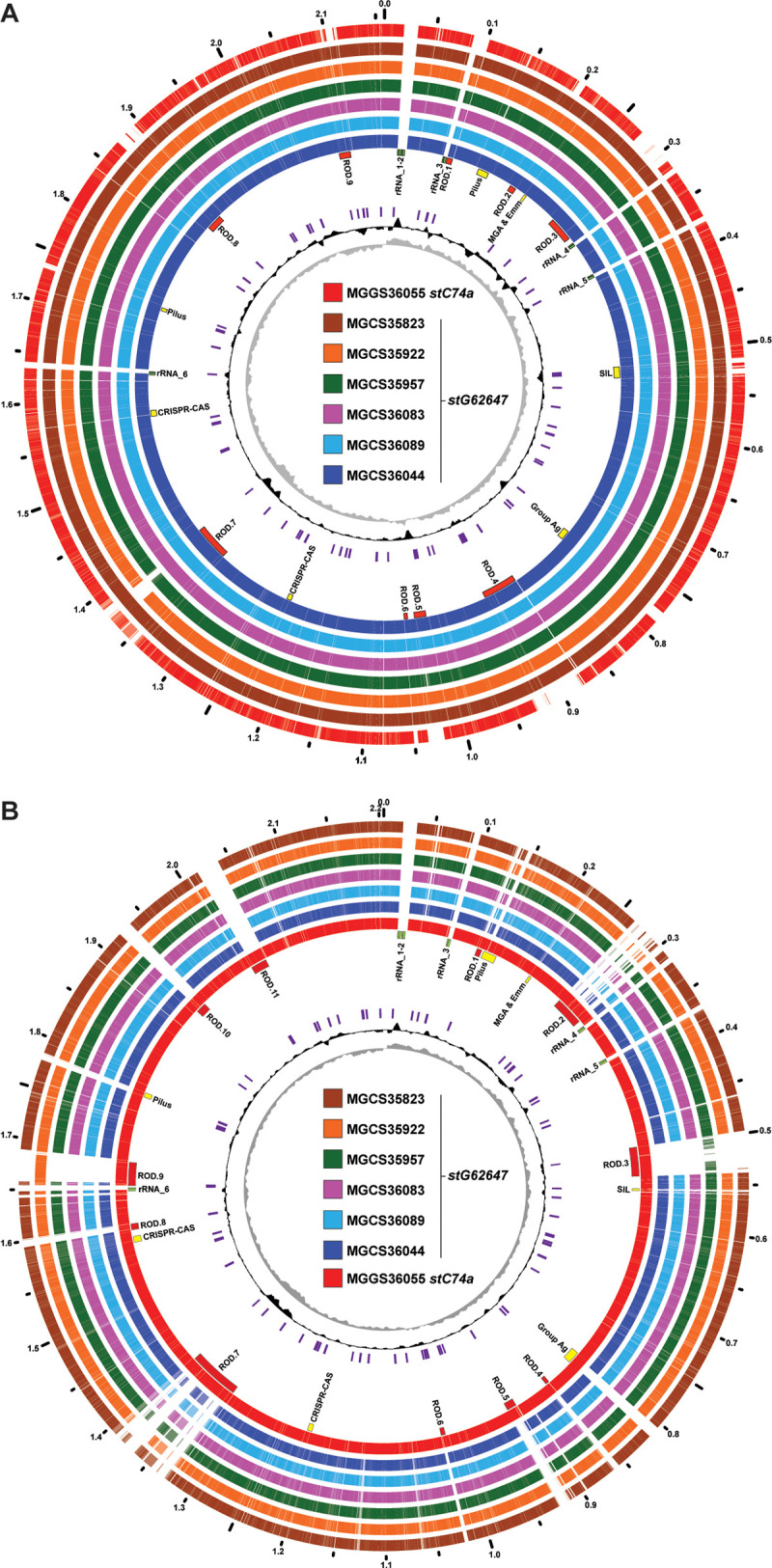
Comparative genome atlases. (A) Illustrated is the 2,158,391-bp genome of the GCS, ST20, *stG62647* strain MGCS36044 and the genetic content it shares with the six other strains of the cohort, as determined by Blastn comparison of CDSs (at 80% identity) relative to the MGCS36044 genome. The common CDS content of the seven strains is shown in the outermost rings, colored as indicated in the central index. The GC skew, indicating the forward and reverse replicores, is plotted in the innermost ring in gray. The G+C percent above and below the genome average (in a sliding window of 10,000 bp) is plotted in the adjacent ring in black. Insertion sequences are shown in purple. Genomic landmarks are indicated as labeled in the ring adjacent to the strain MGCS36044 genome. Of note are nine regions of difference constituting 215.4 kbp, which contain gene content that is largely present in the six *stG62647* genomes but is largely absent from the *stC74a* comparator strain MGGS36055 genome (see Table S3 in the supplemental material). (B) Illustrated is the 2,204,518-bp genome of GGS, ST17, *stC74a* strain MGGS36055 and the genetic content it shares with the six other strains of the cohort. Of note, there are 11 regions of difference constituting 270.7 kbp which contain gene content present in the *stC74a* MGGS36055 genome but largely absent in the six *stG62647* genomes (Table S4). For both strain MGCS36044 and strain MGGS36055, many of the RODs encode an integrase and have a G+C content that is 3% to 7% lower than that of the genome average (approximately 39.4%), consistent with these regions potentially being integrative mobile genetic elements of exogenous origin.

The seven genomes differed in size primarily due to discrete regions of difference (RODs) in gene content attributable to integration of putative mobile genetic elements (MGEs), with gene content characteristic of integrative conjugative elements (ICEs) (47) and phages (48) (Fig. 1). These RODs are the major source of genetic diversity and gene content difference among the seven genomes, constituting on average 267.4 kbp or 12.2% of each genome sequence. In comparing gene content among the seven genomes using ST20/*stG62647* strain MGCS36044 as the reference, there were nine RODs (comprising 215.4 kbp) that had gene content that was largely present among all six of the ST20/*stG62647* genomes but was largely absent from the ST17/*stC74a* strain MGGS36055 genome (Fig. 1A and Table S4). Conversely, using ST17/*stC74a* strain MGGS36055 as the reference, there were 11 RODs (comprising 270.2 kbp) that had gene content that was present in the *stC74a* strain MGGS36055 genome but was largely absent from the six *stG62647* genomes (Fig. 1B and Table S5). Cumulatively, these 20 RODs constituted 485.6 kbp of genetic content differing between strains MGCS36044 and MGGS36055. These RODs ranged in size from 5.2 to 70.7 kbp, and each encoded 4 to 70 inferred genes. Nearly all of these RODs encoded a site-specific integrase or recombinase gene, most of which were terminally located. Moreover, the G+C content of most of these RODs was on average 3% to 7% lower than the overall average of the genome. These characteristics—variable presence among the strains, differing from the core genome in nucleotide composition, and encoding an integrase gene—were all consistent with these RODs being integrative mobile genetic elements that had been acquired exogenously. Although not systematically evaluated, much of the gene content of the RODs shared sequence similarity with MGEs (phages and ICEs) of other streptococci, in particular S. pyogenes and S. dysgalactiae subsp. *dysgalactiae*. The larger (≥30 kbp) RODs had mobilization and structural gene content characteristic of ICEs and phages, whereas many of the smaller RODs encoded an integrase- or recombinase-like gene but lacked other gene content commonly found in ICEs (e.g., genes encoding conjugal transfer proteins) and phages (e.g., genes encoding coat and tail structural proteins), making the nature of their putative mobilization cryptic. These smaller RODs may represent integrative and mobilizable elements (IMEs) (49) or satellite prophages (48). Some of the smaller RODs, which lacked an integrase gene, were flanked terminally by insertion sequences which were prevalent in the S. dysgalactiae subsp. *equisimilis* genomes (average, 95) and may have represented composite transposons.

### An extrachromosomal ICE in *stG62647* strain MGCS35922.

**FIG 2:**
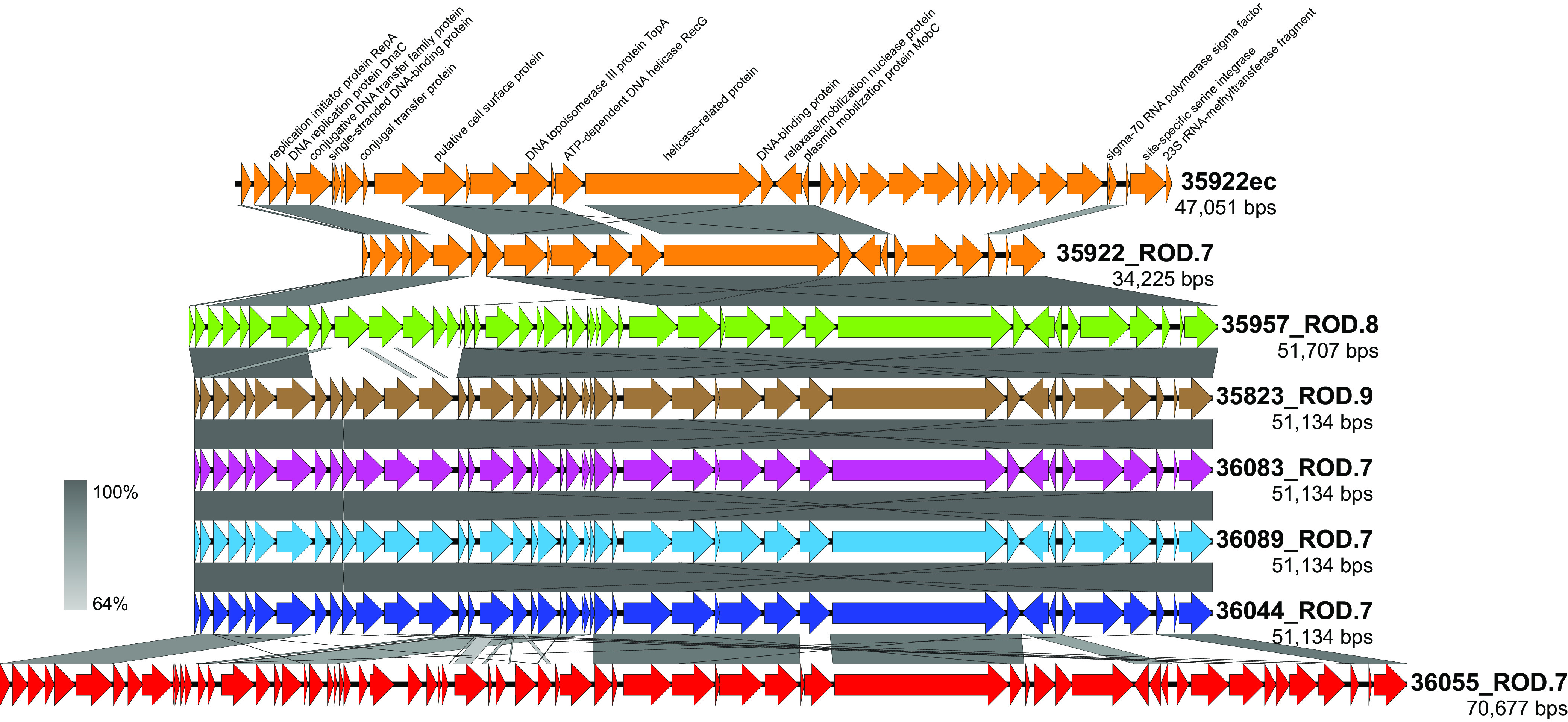
Comparison of the ROD.7 and 35922ec ICEs. Illustrated is an alignment of the 47-kb 35922ec extrachromosomal element in the strain MGCS35922 genome with the ROD.7 equivalent elements of all seven isolates of the cohort. Shown in a gradient of gray, indicating percent sequence identity, are strain-to-strain aligned regions. These elements have conjugal transfer gene content indicating that they are ICEs. All have a PinE superfamily site-specific serine integrase at their 3′ end which directs integration into the 23S rRNA methyltransferase gene *rmlD* (aka *rumA*). The 5′ end of *rmlD* is part of the 35922ec ICE. These ICEs are most closely related among the six *stG62647* strains, being 100% identical in sequence among strains MGCS35823, MGCS36044, MGCS36083, and MGCS36089 throughout their 51.1-kbp lengths, indicating a very recent common ancestor. The ICE in *stC74a* strain MGGS36055 has 52.6% of its sequence in common with that of strain MGCS36044 ROD.7. Most distant is the 35922ec ICE, which has 45.9% of its sequence in common with that of strain MGCS36044 ROD.7.

In addition to the 20 RODs that largely differed in all six *stG62647* strains from *stC74a* strain MGGS36055 and vice versa, there were several additional RODs with gene content largely present in only one *stG62647* strain of the cohort (Table S6). Among these strain-specific RODs, we identified a 47-kb circular element that Unicycler assembled in its entirety extrachromosomally from the *stG62647* strain MGGS35922 closed genome. Because of the concern that this might represent an assembly error, the strain MGGS35922 extrachromosomal (35922ec) circularized element was checked for consistency with the short- and long-read sequencing data by read mapping. No locus was found where there was discontinuity in either the paired mapping of short reads or disrupted mapping of long reads to indicate that the element was inappropriately assembled as an extrachromosomal closed circularized sequence. Moreover, 35922ec had gene content encoding conjugal transfer and mobilization proteins and a serine-type site-specific integrase or recombinase flanked by an attachment site sequence homologous to (i.e., targeting integration to) the chromosomally encoded 23S RNA methyltransferase gene *rlmD*, all characteristics consistent with it being an ICE (50) (Fig. 2). Additionally, 35922ec encoded a RepA-like replication protein and a chromosome segregation ATPase protein, findings consistent with the capacity for episomal replication (47). Compared to the seven closed genomes, the 35922ec ICE had sequence similarity with elements integrated at *rmlD* and corresponding to ROD.7, as defined relative to the strain MGCS36044 genome (Fig. 1A) and strain MGGS36055 genomes (Fig. 1B). 35922ec had partial gene content that was present in the genomes of all seven strains of the cohort, including the MGGS35922 genome (Fig. 2). As an additional test of the genome assembly validity, PCR was used to check the structure at the putative extrachromosomal element 35922ec putative attachment site and chromosomal attachment and integration sites flanking 35922_ROD.7 (Fig. S1). All PCR amplicons were consistent with the genome architecture produced by the Unicycler hybrid sequencing assembly. 35922ec and 35922_ROD.7, although having some ICE-like gene content in common, differed in sequence and overall length by 13 kbp. We hypothesized that the 35922ec ICE did not integrate into the strain MGGS35922 chromosome because the *rlmD* integration site was already occupied by 35922_ROD.7 ICE (Fig. 2). Experimental studies are required to test this hypothesis.

### Additional sources of genetic content diversity.

**FIG 3:**
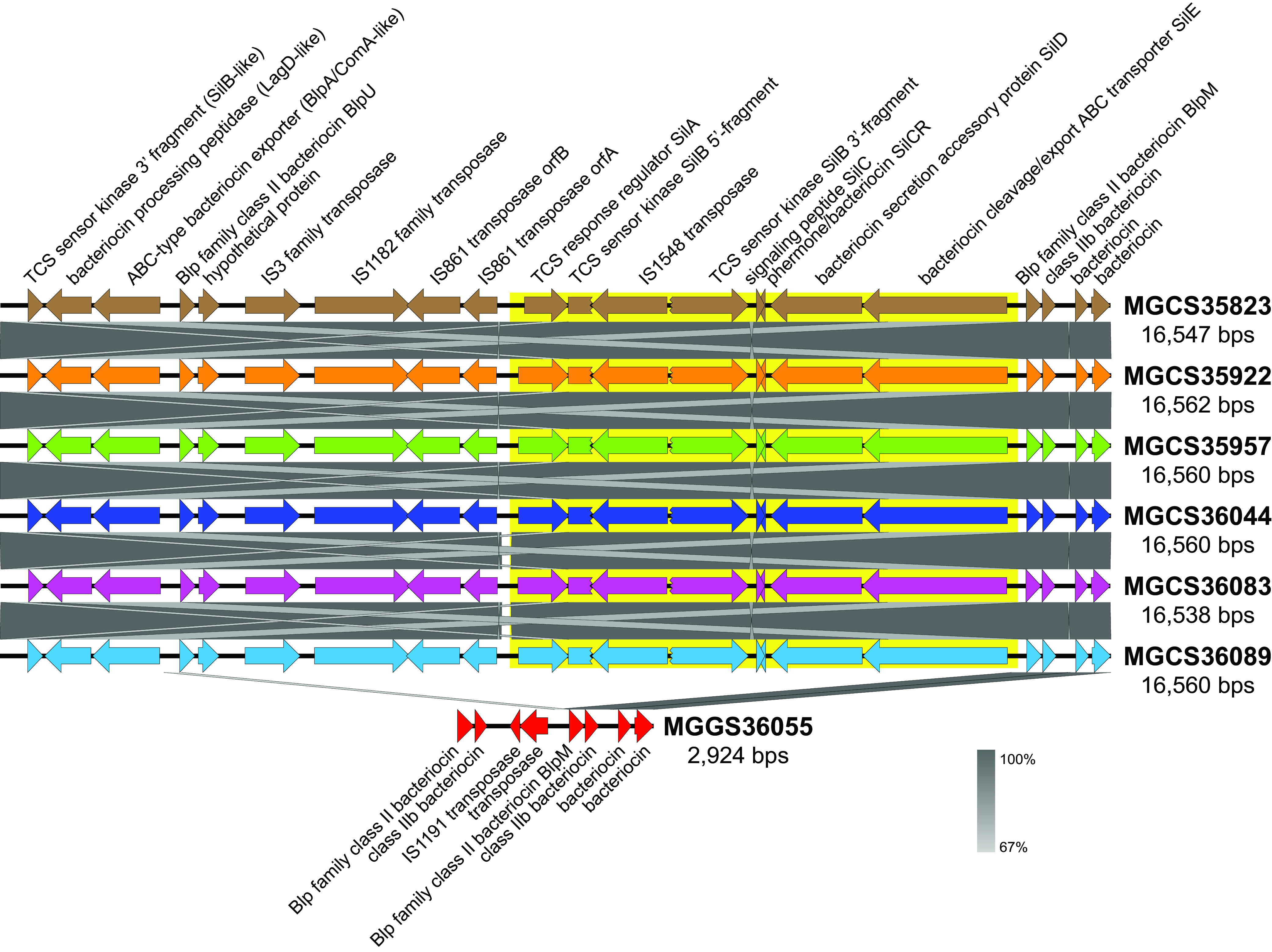
Variation in the streptococcal invasion locus. Illustrated is an alignment of the streptococcal invasion locus between all seven isolates of the cohort. Shown in a gradient of gray, indicating percent sequence identity, are strain-to-strain aligned regions. The SIL is composed of six genes: *silA*, *-B*, *-C*, *-CR*, *-D*, and -*E* (highlighted in yellow), which are variably present among S. dysgalactiae subsp. *equisimilis* genomes. In multiple streptococcal species, the SIL genes are flanked by additional variably present genes with similarity to genes involved in competence quorum sensing and bacteriocin production. Together, the SIL and flanking competence/bacteriocin-like genes constitute the extended SIL. The extended SIL among the six ST20/*stG62647* genomes is 16.5 kb, encodes 21 genes which, strain to strain, share greater than 99.4% nucleotide identity. SilB is disrupted by insertion of IS*1548* in all six *stG62647* genomes. In contrast, the corresponding locus of the ST17/*stC74a* strain MGGS36055 genome is only 2.9 kbp and lacks all six SIL genes. Sequence similarity between *stC674a* strain MGGS36055 and the six *stG62647* strains is limited primarily to the four bacteriocin genes at the 3′ end of the extended SIL.

In addition to the RODs with characteristics of MGEs, there were several additional regions of lower variation in genetic content that differed between the six *stG62647* strains and *stC74a* strain MGGS36055. These regions included gene content encoding pilus, M protein, carbohydrate group antigen, and the extended streptococcal invasion locus (SIL) (each indicated as a landmark in Fig. 1). These were not regions of complete gene content difference. That is, they did not simply differ by strain-to-strain gene presence or absence, but rather had partial gene content that was conserved and partial content that varied. The regions had in common that each encoded one or more inferred secreted or surface-attached molecules. In principle, these proteins and sugar polymers interact with the host immune system, and genetic variation at these loci may reflect diversifying evolutionary selection for polymorphisms that contribute to evasion of the host immune response. Oppegaard et al. (9) reported that among the 18 ST20/*stG62647* isolates collected in western Norway that they studied, the SIL quorum-sensing regulon (51–53) was disrupted by insertion of IS*1548* into the *silB* gene encoding the two-component system sensor histidine kinase SilB. The investigators postulated that disruption of the *silB* gene contributed to ST20/*stG62647* isolates causing severe infections. All six of the *stG62647* isolates collected in French Brittany that we studied also had the same transposon insertion (IS*1548*) disrupting *silB*, whereas *stC74a* strain MGGS36055 lacked all six SIL genes (*silA*, -*B*, -*C*, -*CR*, -*D*, and -*E*) (Fig. 3). Among the 21 closed S. dysgalactiae subsp. *equisimilis* genomes in GenBank, the SIL genes are absent in 5 but present in 16 (Table S3). However, in 5 of the 16 genomes with the SIL genes present, one or more of the SIL genes were disrupted, including *silB* in the strain TPCH-A19. Clearly the SIL region, not to mention the additional bacteriocin-like gene content flanking it and constituting the extended SIL, was fairly variable among S. dysgalactiae subsp. *equisimilis* genomes. The function of SIL has not been experimentally characterized in S. dysgalactiae subsp. *equisimilis*, and thus the genes it regulates and its role in S. dysgalactiae subsp. *equisimilis* pathogenesis are currently unknown.

### Phylogenetic analysis of the seven genomes.

**FIG 4:**
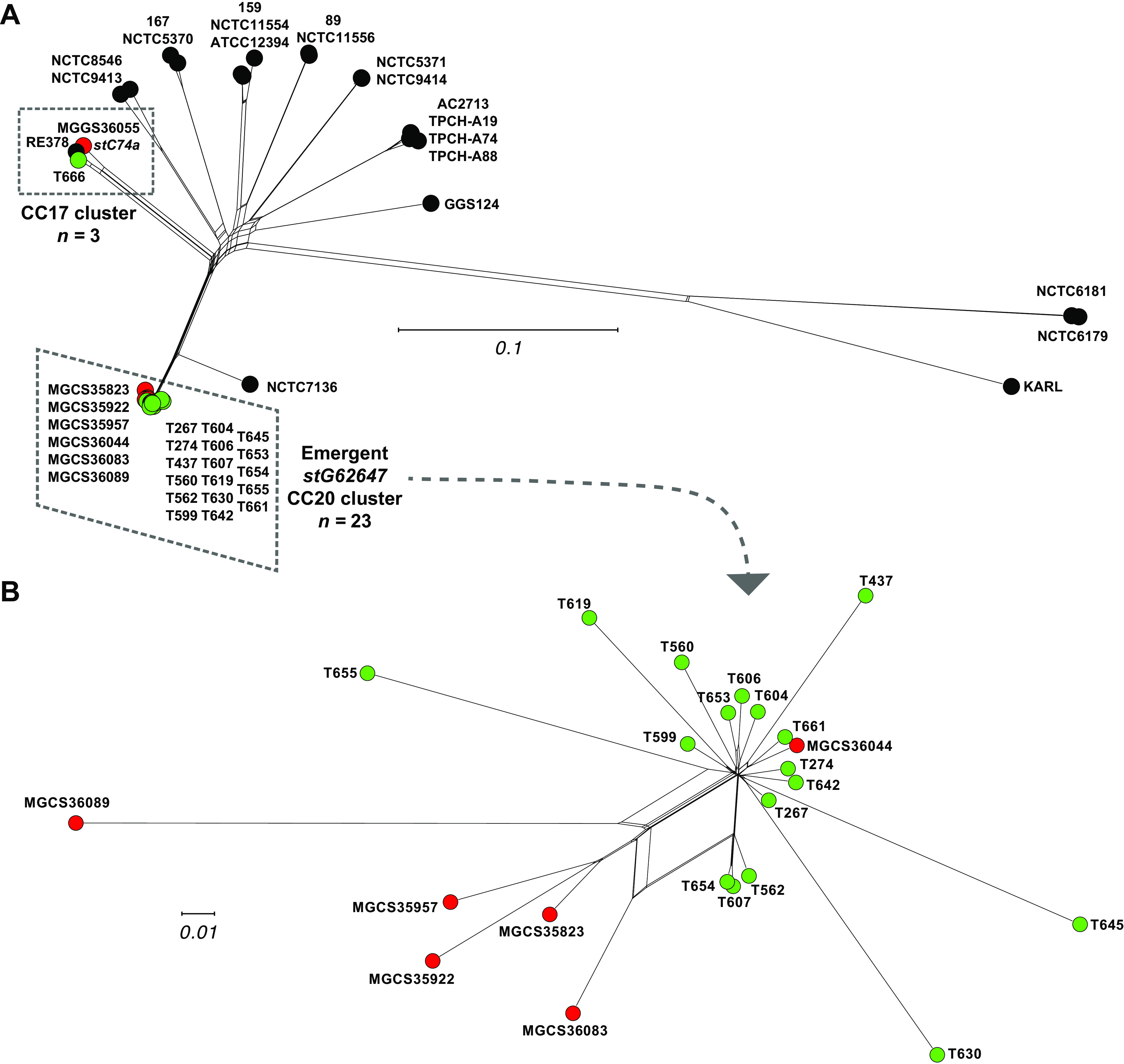
(A) Genetic relationships among 46 S. dysgalactiae subsp. *equisimilis* isolates inferred by neighbor-network based on 84,926 core chromosomal SNPs. Illustrated are three strain sets: 21 closed reference genomes from the National Center for Biotechnology Information (NCBI) Microbial Genome Database (MGDB) are in black, 18 *stG62647* isolates collected in Norway are in green (whole genome sequenced by Oppegaard et al.), and seven isolates (six *stG62647* and one *stC74a*) collected in French Brittany are in red. (B) Genetic relationships among 23 (17 Norway and 6 French Brittany) clustered and closely related S. dysgalactiae subsp. *equisimilis stG62647* isolates of human infections inferred by neighbor-network based on 3,369 core chromosomal SNPs. The strain-to-strain mean genetic distance (MGD, i.e., core SNP difference) between the 21 closed reference genomes is 20,428 (range,137 to 42,147), the MGD between the 17 Norway emergent *stG62647* cluster isolates is 291 (range, 42 to 776), the MGD between the 6 French Brittany *stG62647* closed genomes is 495 (range, 237 to 847), the MGD between all 23 isolates of the emergent *stG62647* genetic cluster is 379 (range, 42 to 1,080), and the MGD between the 6 French Brittany *stG62647*/ST20 isolates and *stC74a*/ST17 strain MGGS36055 is 11,022 (range, 10,976 to 11,097).

Phylogenetic analysis of the seven genomes based on core-genome single-nucleotide polymorphisms (SNPs) found that the six *stG64647* strains we studied were closely genetically related, differing from each other on average by only 495 core chromosomal SNPs (Fig. 4). These six isolates (collected in Brittany, France between 2015 and 2018) were of the same multilocus sequence type clonal complex, CC20 (CC20 is composed of ST20 and its single-locus variants), as were 18 of the 19 *stG62647* strains recently reported by Oppegaard et al. (9) as an emergent cause of severe infections in western Norway. Phylogenetically, these two sets of isolates clustered together (Fig. 4), consistent with them being clonally derived from an evolutionarily recent common progenitor. In contrast, the *stC74a* comparator strain MGGS36055 along with reference strain RE378 and Norway isolate T666 were multilocus sequence type CC17 and differed on average from the CC20 isolates by 12,050 core SNPs (Fig. 4).

### Lancefield carbohydrate group identification.

S. dysgalactiae subsp. *equisimilis* strains can be of streptococcal carbohydrate group antigen types G, C, or A. Bioinformatic analysis of the region of the genome encoding the Lancefield carbohydrate group antigen synthesis genes of the six clonally related *stG62647* strains indicated that these organisms are group C and that the *stC74a* strain MGGS36055 is group G. This bioinformatic determination was confirmed experimentally with a commonly used immunologic assay, as described in Materials and Methods (Table 1).

### MICs for beta-lactam antibiotics.

Decreased susceptibility of beta-hemolytic streptococci to beta-lactam antibiotics has been reported with increased frequency in recent years (3, 16, 39, 54–58). Concerningly, Fuursted et al. (16) described four clonally related S. dysgalactiae subsp. *equisimilis* strains resistant to penicillin *in vitro* and reported that these strains had multiple amino acid changes in PBP2X, the primary target of penicillin in streptococci. Thus, we evaluated MICs in our seven S. dysgalactiae subsp. *equisimilis* strains for five commonly used beta-lactam antibiotics, including amoxicillin, cefotaxime, cefoxitin, meropenem, and penicillin G. None of the seven S. dysgalactiae subsp. *equisimilis* strains had MICs deviating from the susceptible range for the five beta-lactam antibiotics tested (Table 2). Consistent with our *in vitro* susceptibility data, the PBP2X gene in the seven isolates studied did not have amino acid changes expected to result in substantial increases in MICs to these antibiotics. That is, there were no amino acid changes in the PBP2X transpeptidase domain and catalytic motifs that have been associated with reduced beta-lactam susceptibility in streptococci (data not shown).

**TABLE 2:** Beta-lactam antibiotic MICs for the seven S. dysgalactiae subsp. *equisimilis* strains studied

Strain	MIC (mg/liter)				
	Amoxicillin	Cefotaxime	Cefoxitin	Meropenem	Penicillin G
MGCS35823	0.032	0.023	0.75	0.012	0.012
MGCS35922	0.032	0.023	1.00	0.008	0.012
MGCS35957	0.032	0.023	0.50	0.008	0.012
MGCS36044	0.032	0.023	1.00	0.012	0.016
MGCS36083	0.032	0.032	1.00	0.012	0.016
MGCS36089	0.016	0.023	1.00	0.012	0.012
MGGS36055	0.016	0.023	0.75	0.006	0.012

### *stG62647* strains are significantly more virulent than a genetically divergent strain of *emm* type *stC74a*.

**FIG 5:**
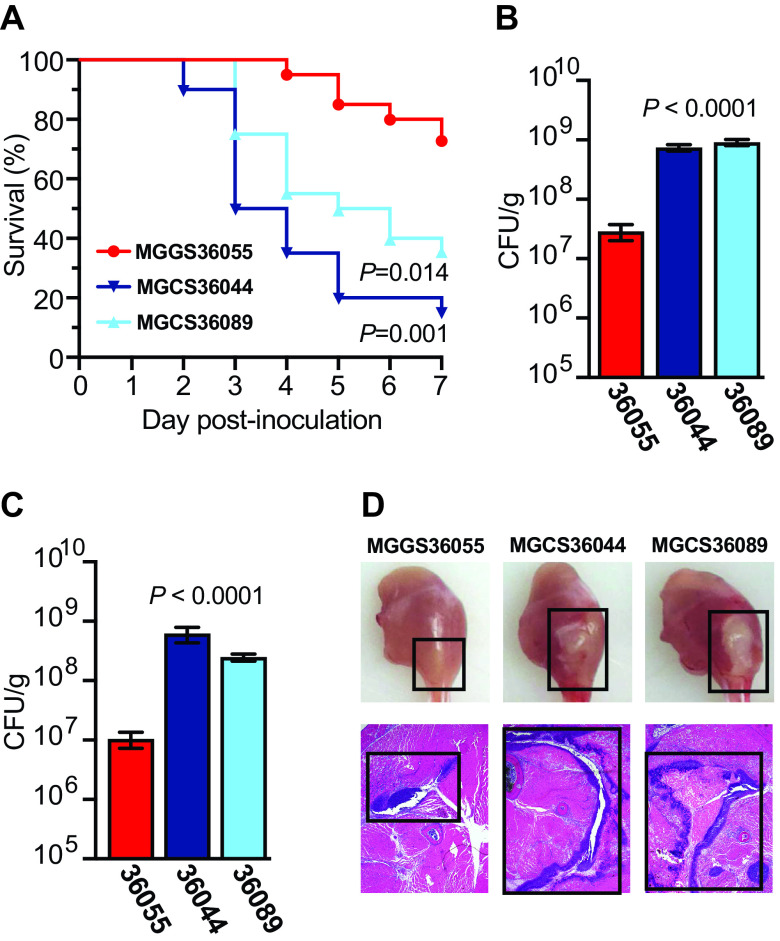
Virulence assessment of *emm* type *stG62647* strains MGCS36044 and MGCS36089 and *stC74a* strain MGGS36055 in a mouse model of necrotizing myositis. (A) Kaplan-Meier survival curves are shown. Survival was monitored for 7 days (log-rank test, *n *= 20 mice per strain). (B and C) CFU recovery from infected limbs was performed on day 1 and day 2 after infection. Replicate data are expressed as means ± SEM (Kruskal-Wallis test, *n *= 20 mice per strain). (D) Gross and microscopic examination of infected limbs on day 2 postinfection. Lesion size and necrotic tissue are highlighted (black boxes).

Epidemiological surveillance reports and outbreak investigations from diverse locations suggest that *emm* type *stG62647* strains may cause more virulent human infections than S. dysgalactiae subsp. *equisimilis* strains of other *emm* types, but this has not been experimentally shown. To test the hypothesis that *emm* type *stG62647* strains of the ST20 genetic lineage are more virulent than other S. dysgalactiae subsp. *equisimilis* genetic lineages that cause abundant human infections, we compared the virulence of ST20/*stG62647* strains MGCS36044 and MGCS36089 relative to that of ST17/*stC74a* strain MGGS36055 using a well-established mouse model of necrotizing myositis (38–41, 59). These three selected strains were of genetic lineages that cause abundant infections and were also temporally, geographically, and disease manifestation matched. The strains were recovered between March and June 2018 from cases of invasive osteitis infection that occurred in Brittany, France. Of note, although *stG62647* strains MGCS36044 and MGCS36089 were virtually identical in gene content, they differed by 755 core chromosomal SNPs, which was greater than the average of 379 SNPs that separated the 23 strains of the emergent *stG62647* cluster (Fig. 4), consistent with MGCS36044 and MGCS36089 having an evolutionarily less recent common ancestor. Compared to *stC74a* strain MGGS36055, the two *stG62647* strains, MGCS36044 and MGCS36089, led to significantly higher bacterial burdens in infected limbs and caused significantly more mortality (Fig. 5A to C). The *stG62647* strains also caused significantly larger lesions with more tissue destruction (Fig. 2D). Together, these data demonstrated that the two *stG62647* strains studied were significantly more virulent than the comparator *stC74a* strain studied and supported the contention (9) that strains of the emergent ST20/*stG62647* clonal lineage cause infections with a more aggressive clinical course.

The data presented here show that the six Brittany, France strains of type ST20/*stG62647* we studied were clonally derived. These are the only isolates of ST20/*stG62647* for which genomes have been sequenced to closure. Analogous to important findings presented by Oppegaard et al. (9) for strains from western Norway, members of this clone also have IS*1548* inserted in the *silB* open reading frame. We also demonstrated that members of this clone have enhanced virulence in a commonly used mouse model of serious invasive disease, thereby adding new information about this emergent clone. However, our study was not designed to address the important topics of the molecular genetic events underlying emergence of the ST20/*st62647* clone or the molecular mechanisms bearing on enhanced virulence of members of this clone. Our findings underscore the need for substantially expanded genomics and molecular pathogenesis investigations of S. dysgalactiae subsp. *equisimilis* strains causing human infections.

## MATERIALS AND METHODS

### Strains.

The seven strains analyzed were part of a comprehensive prospective study of S. dysgalactiae subsp. *equisimilis* human infections conducted from 2010 through 2018 in Brittany, France (33, 60). The study was conducted similarly to an investigation of S. pyogenes infections in Brittany, France (60) and will be described in detail elsewhere. Briefly, S. dysgalactiae subsp. *equisimilis* isolates were identified by standard diagnostic procedures used in the clinical microbiology laboratory, University Hospital of Rennes, France. Culture diagnosis was also conducted at the University Hospital, Rennes, France and analyzed by matrix-assisted laser desorption ionization–time of flight mass spectrometry (Bruker Daltonics GmbH, Germany). Isolates were subcultured at 37°C with 5% CO_2_ on Columbia blood agar plates containing 5% sheep blood (Bio-Rad, France) and stored at −80°C.

### Whole-genome sequencing.

The genomes of the S. dysgalactiae subsp. *equisimilis* strains were sequenced with methods described previously for S. pyogenes (17, 38, 54, 61, 62). Briefly, strains were grown at 37°C with 5% CO_2_ on tryptic soy agar with 5% sheep blood (Becton, Dickinson, Franklin Lakes, NJ) or in Todd-Hewitt broth with 2% (wt/vol) yeast extract (THY; Difco Laboratories, Franklin Lakes, NJ). Chromosomal DNA for Illumina short-read sequencing was isolated with the RNAdvance viral kit (Beckman Coulter, Brea, CA) and a BioMek i7 instrument (Beckman Coulter). Libraries were prepared with the NexteraXT kit (Illumina, San Diego, CA) and sequenced with a NovaSeq instrument (Illumina) using a 2 × 250-bp protocol. Chromosomal DNA for long-read sequencing was isolated with a DNeasy blood and tissue kit (Qiagen, Germantown, MD). Libraries were prepared with either a native barcoding kit or rapid barcoding kit (Oxford Nanopore Technologies, United Kingdom) and sequenced with a GridION instrument using version R10.4 or R9.4.1 flow cells (Oxford Nanopore Technologies), respectively.

### Genome assembly, closure, and annotation.

Illumina paired-end short reads were base call error corrected with Karect (63), and Oxford Nanopore long reads were corrected with the FM index long read corrector (64). Hybrid assemblies of the preprocessed short- and long-read sequencing were generated with Unicycler (65). Unitigs from genomic assemblies that did not close were ordered against a closely related reference genome (or closed assembly) with progressiveMauve (66). Gaps between the ordered unitigs (mostly rRNA operons) were manually spanned or closed by splicing in corresponding sequences from one or more closely related closed genomes with Sequencher (Gene Codes, Ann Arbor, MI). These closed draft genome assemblies were checked for consistency with the Illumina PE short-read sequencing data by read mapping with SMALT (67) coupled with polymorphism detection with FreeBayes (68) and for structural inconsistencies with NucBreak (69). Consistency between the closed draft assemblies and the Oxford Nanopore long-read sequencing data was checked by read mapping with MiniMap2 (70). Correspondence of the mapped short and long reads with the draft closed genomes was visually inspected with Tablet (71). This reference-assisted and -directed genome closure process was iterated until discrepancies between the sequence data and the draft genomes were resolved. The resultant finished genomes were annotated *de novo* with RAST (72) and via transfer using PROKKA with S. dysgalactiae subsp. *equisimilis* strain ATCC 12394 as the reference (73). Annotations were merged and manually curated with Artemis (74). Multilocus sequence types of the isolates were determined by BLAST relative to the PubMLST database (https://pubmlst.org/), *emm* types were determined relative to the CDC *emm* type database (https://www2.cdc.gov/vaccines/biotech/strepblast.asp), and insertion sequence types were determined relative to the ISfinder database (https://isfinder.biotoul.fr/). Comparative genome atlases were generated with GView (75), and sequence alignment comparisons were generated with EasyFig (76).

### Mouse model of necrotizing myositis and CFU determination.

The mouse model of necrotizing myositis used in these experiments has been previously described and used extensively (38–41, 59). Animals were infected intramuscularly in the right lower hindlimb with 1 × 10^8^ CFU of the indicated strain and monitored daily for near mortality using standard criteria (77). Survival was graphed as a Kaplan-Meier curve, and statistical differences were determined with a log-rank test (GraphPad Prism V9.4.1; San Diego, CA). CFU from infected muscle were determined by culturing limb homogenates as described elsewhere (38–41, 59). Briefly, each infected limb was amputated, weighed, homogenized (Omni International, Kennesaw, GA) in 1 mL sterile phosphate-buffered saline, serially diluted, plated, and cultured overnight. CFU data were graphed as means ± standard errors of the means (SEM), and statistical differences were determined with a Kruskal-Wallis test. For histopathology examination, amputated limbs were photographed with a Solo8 Hovercam (Pathway Innovations, Las Vegas, NV) and processed with standard pathology laboratory methods.

**(i) Ethics statement.** Mouse experiments were approved by the Institutional Animal Care and Use Committee of Houston Methodist Research Institute (protocol IS00006169).

### Identification of Lancefield group carbohydrate.

The Lancefield carbohydrate group antigen of the seven strains studied was determined with a latex agglutination method (BBL Streptocard enzyme latex test; Becton, Dickinson, Franklin Lakes, NJ) on overnight growth harvested from blood agar plates.

### Determination of MICs for antimicrobial agents.

MICs for beta-lactam antibiotics, including amoxicillin, cefotaxime, cefoxitin, meropenem, and penicillin G, were determined by the MIC test strip method (Liofilchem, Waltham, MA) according to the manufacturer’s instructions. Strains were grown overnight on tryptic soy agar with 5% sheep blood (Becton, Dickinson), and fresh colonies were collected with the BBL prompt inoculation system (Becton, Dickinson) and plated onto Mueller-Hinton agar (Remel Microbiology Products, Lenexa, KS) with the antibiotic test strips. MICs (in milligrams per liter) were read after 24 h of incubation independently by two technologists.

### Data availability.

The annotated closed genome assemblies and short- and long-read sequencing data for the seven S. dysgalactiae subsp. *equisimilis* strains studied have been submitted to the National Center for Biotechnology Information (NCBI) under Bioproject accession number PRJNA925803.
