# Supplementary material for: Analysis of the Genomics and Mouse Virulence of an Emergent Clone of Streptococcus dysgalactiae Subspecies equisimilis
Source: Microbiol Spectr. 2023 Mar 27;11(2):e04550-22. doi: 10.1128/spectrum.04550-22 (PMC10100674; doi:10.1128/spectrum.04550-22)
Supplement: Supplemental file 1 — Figure S1. Download spectrum.04550-22-s0001.pdf, PDF file, 0.1 MB [file spectrum.04550-22-s0001.pdf]

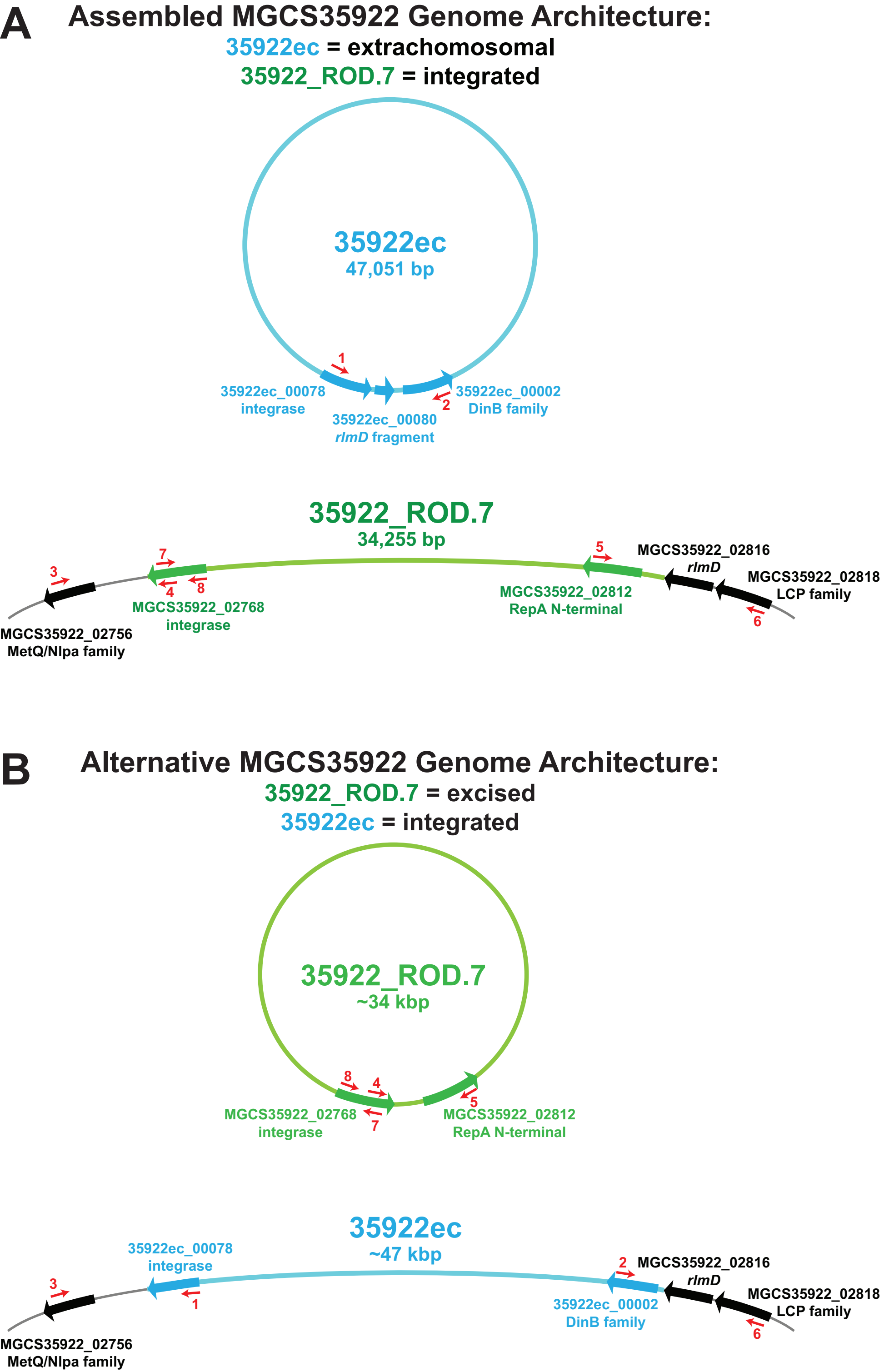

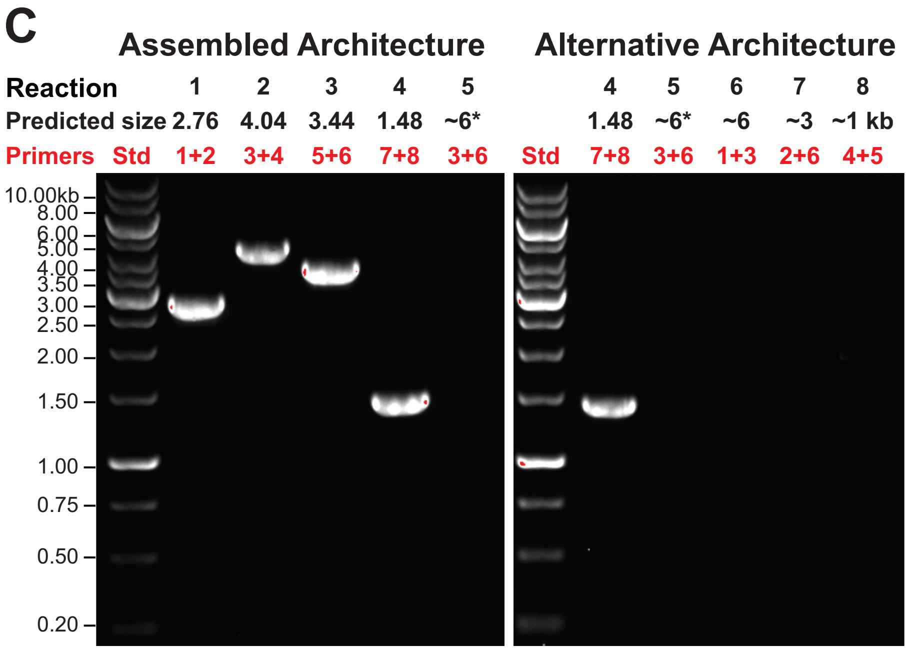

*An amplicon is expected only if the *rlmD* integration site is vacant.

Fig. S1. PCR analysis of MGCS35922 genome architecture. **(A)** Assembled architecture. Illustrated is the genome architecture as produced by hybrid sequencing assembly with Unicycler. A closed circular sequence of 47.1 kbp assembled extrachromosomally and was designated 35922ec (shown in blue). The chromosome assembled as a closed circular sequence of 2.22 Mbp with a region of variably present gene content (i.e. not conserved core gene content) of 34.3 kb integrated into the chromosome adjacent to the rRNA methytransferase gene *rlmD*. This SDSE genome-to-genome region of difference in gene content was designated 35922_ROD.7 (shown in green). Both 35922_ROD.7 and 35922ec have gene content encoding for conjugal transfer and site-specific integration/recombination and are likely integrative-conjugative elements (ICEs). **(B)** Alternative architecture. Illustrated is a hypothesized alternative architecture for the MGCS35922 genome. Given that ICEs are MGEs capable of reversible integration into and excision from the chromosome, the alternative architecture is predicated on 35922_ROD.7 being extrachromosomal (i.e. having excised from the chromosome) and 35922ec being chromosomal (i.e. having integrated into the chromosome). **(C)** Illustrated are PCR amplicons based on the assembled and hypothesized alternative architectures for MGCS35922 genome. Reactions 1-3 test for consistency with the Unicycler assembled architecture, and reactions 6-8 test for the hypothesized alternative architecture. Reaction 4 is a positive control that is expected to amplify independent of the genome architecture, if the 35922_ROD.7 integrase gene (locus tag MGCS35922_02768) is present. Reaction 5 is a negative control, under the PCR conditions used, an amplicon is only expected if there is no MGE integrated adjacent to the *rlmD* gene (locus tag MGCS35922_02894). All amplicons are consistent with the Unicycler assembled genome architecture. The failure of reaction 5 (primers 3+6) to produce an amplicon under conditions in which other amplicons of ~4 kb did amplify (primers 3+4 and 5+6), is consistent with the ICE integration site adjacent to *rlmD* being stably occupied. Primer sequences used are provided in supplemental table S7.
